# Comparison of Different Types of Poly-L-Lactic Acid Microspheres In Vitro and In Vivo Studies

**DOI:** 10.1093/asjof/ojae091

**Published:** 2024-10-22

**Authors:** Ruilin Liu, Tong He, Ruizhi Li, Shiwei Wang, Chen Lai, Kun Zhang

## Abstract

**Background:**

Biodegradable polymers are commonly used as dermal fillers in plastic surgery. Among these, poly-L-lactic acid (PLLA) distinguishes itself owing to its good biocompatibility, degradability, and ability to act as a collagen stimulator.

**Objectives:**

In this study, the differential behavior of PLLA microspheres with varying microscopic morphology and surface hydrophilicity was investigated both in vitro and in vivo.

**Methods:**

The introduction of short hydrophilic polyethylene glycol (PEG) chains into the PLLA molecule was employed to modify the morphology and enhance the surface hydrophilicity of the microspheres. The morphology and physicochemical properties of the PLLA and PLLA-b-PEG microspheres were characterized. Irregular PLLA particles, PLLA, and PLLA-b-PEG microspheres were implanted into the subcutaneous tissue of rabbit models, and at 4, 26, and 52 weeks after implantation, biopsy samples were collected for hematoxylin and eosin and Masson’s trichrome staining to evaluate differences in the tissue response between different implants.

**Results:**

The results of in vitro research demonstrated that while the addition of short-chain hydrophilic PEG afforded a smoother surface for the microspheres, it had no significant effect on the molecular weight and degradation rate of PLLA. The histological examination revealed that the PLLA-b-PEG microspheres exhibited enhanced biocompatibility compared with the pure PLLA microspheres, while the irregular PLLA particles showed the highest inflammatory response among the 3 materials.

**Conclusions:**

In this study, we found that the properties of PLLA were improved upon modification by short-chain PEG without reducing the collagen regeneration ability, thereby affording a better histocompatibility.

**Level of Evidence: 5 (Therapeutic):**

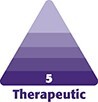

Skin aging is caused by both internal and external factors. In addition to external factors, such as repeated exposure to ultraviolet radiation, immunological responses to tissue degeneration and damage are the major internal contributors to skin aging.^1,2^ Despite the body's attempt to promote tissue repair during this process, it still necessitates external interventions for effective antiaging outcomes.^3,4^ To decelerate, halt, or potentially reverse the aging process, medical practitioners are currently using aesthetic medical methods, especially minimally invasive methods,^5^ to rejuvenate the structural and functional integrity of aging tissues by intervening in the process of the skin's immunological responses, such as microdermabrasion, microneedle therapy, chemical peel, laser skin resurfacing, micro-focused ultrasound, radiofrequency skin tightening, botulinum toxin, and dermal fillers.^6-11^

Poly-L-lactic acid (PLLA) is an emerging dermal filling material capable of inducing collagen formation at the injection site.^12^ The local filling effect can last up to 2 years, eliminating the limitations associated with sodium hyaluronate filling products that are commonly used in face filling.^13^ However, the in vivo environment has a high water content, which makes the hydrophobic polymer PLLA poorly compatible with the body.^14^ Therefore, PLLA can induce stimulation in the physiological environment, which in turn can trigger inflammation and adverse reactions, although inflammation increases collagen levels.^15^ The analysis of the US FDA manufacturer and user facility device experience database from various time periods consistently demonstrated that nodule formation was the most common complication associated with PLLA.^16-18^ Nodules arising early are likely to be technique dependent, and the risk can be minimized through technical optimization, such as increasing dilution volume and implementing posttreatment massage. Conversely, delayed-onset nodules and granulomas can be due to chronic inflammation responses and represent the most prevalent long-term complications of PLLA. Management of these complications requires intralesional and/or systemic corticosteroids, intralesional 5-fluorouracil, surgical excision, etc. To avoid the irritation caused by PLLA in vivo and reduce the risk of inflammation and adverse reactions, modifying the morphology of PLLA by introducing hydrophilic segments in the PLLA main chain, such as polyethylene glycol (PEG), is a good choice.^19^ The introduction of PEG by forming the block copolymer PLLA-b-PEG changes the surface hydrophilicity of PLLA microspheres. Meanwhile, since PLLA microspheres generally adopt an oil-in-water system during the preparation process, PEG can form a hydrophilic layer at the oil–water interface to help the PLLA oil-phase solution be uniformly dispersed in the aqueous phase, which may further improve the morphology of the microspheres.^20-22^

In this study, we investigated the surface morphologies of both PLLA and PLLA-b-PEG microspheres and evaluated their safety and efficacy in terms of their physiological characteristics and ability as collagen stimulators through in vivo implantation experiments. These efforts will provide valuable theoretical insights into clinical treatments.

## METHODS

### Materials

PLLA (Zhongxing Meiyuan Biotech. Co., Ltd, Chengdu, Sichuan, China) was a commercial product for purchase, PLLA-b-PEG was obtained by ring-opening polymerization of L-lactide with poly(ethylene glycol) methyl ether (mPEG) as the initiator by the Beijing Engineering Lab of Neo-Biodegradable Materials (Beijing, China). PLLA microspheres (*M*_w_ = 95 kDa), polydispersity index (PDI = 1.9), and PLLA-b-PEG microspheres (*M*_w_ = 98 kDa; PDI = 1.5) were prepared using the oil–water emulsification method and supplied by the Beijing Engineering Lab of Neo-Biodegradable Materials (Beijing, China). Irregularly shaped PLLA particles, prepared using the freeze-crushing method, were also supplied by the Beijing Engineering Lab of Neo-Biodegradable Materials.

### In Vitro Studies

From April to December 2023, in vitro research experiments have been studied, which are composed of microscopic morphology, physicochemical properties, and their uniformity of distribution.

#### Fourier Transform Infrared Spectroscopy

Fourier transform infrared spectroscopy (FTIR; Thermo Fisher Nicolet 6700) was used to analyze the functional groups of the PLLA and PLLA-b-PEG microspheres.

#### Scanning Electron Microscopy

Trace samples of the PLLA and PLLA-b-PEG microspheres were directly adhered to the conductive adhesive, respectively, and sprayed for 45 seconds at 10 mA. Subsequently, the sample morphology was studied using scanning electron microscopy (SEM; using TESCAN MIRA LMS, Czech) at an acceleration voltage of 2 kV, with the SE2 secondary electron detector employed for image acquisition.

#### In Vitro Degradation

Initially, 0.36 g of dried microspheres (recorded as *m*) was accurately weighed and placed in a glass bottle. Subsequently, 1 mL of HCl solution was added. The glass bottles were then sealed, and the samples were stored at 100 °C ± 2 °C for 2, 4, and 8 h, followed by the addition of 2 mL of NaOH solution for neutralization. Two pieces of medium-speed filter paper (dried at 100 °C ± 2 °C for >15 h) were taken and weighed accurately (recorded as *m*_1_). The sample solution was filtered using these pieces of filter paper by washing with >300 mL of water. The filtered sample and filter paper were dried at 100 °C ± 2 °C for >15 h and then weighed accurately (recorded as *m*_2_).

The degradation rate (*ω*) was determined as follows:


$$\omega =\frac{m-(m_{2}-m_{1})}{m}\times 100\text{\%}$$

#### Characterization of the Dispersion Uniformity of PLLA-b-PEG Microspheres

PLLA-b-PEG microspheres were suspended and dispersed in the sodium hyaluronate gel. Based on the principle that the densities of microspheres and gel are different, X-ray computed tomography (CT; Phoenix GeV tome S240, with a resolution of 0.869 μm) was used to characterize the dispersion uniformity of microspheres in the gel.

### In Vivo Studies

From November 2022 to November 2023, the in vivo studies were conducted. Specifically, it can be divided into the following 3 steps.

#### Animals

Twelve female Japanese white rabbits, aged 4 months and weighing 2.2 to 2.5 kg, were acquired from the Beijing Longan Experimental Animal Breeding Center (Beijing, China). These rabbits were maintained under optimal conditions at a controlled temperature of 23 °C ± 3 °C, relative humidity of 40% to 70%, and subjected to a 12 h light/12 h dark cycle. They were also provided with purified food and water. Following a 1 week acclimatization period, the rabbits were randomly assigned to 3 groups based on different sampling time points. All the experimental procedures involving animals were conducted in accordance with guidelines approved by the Ethics Committee of Beijing Yongxin KangTai Technology Development Co., Ltd (IACUA No. YXKT2022L020).

#### Experimental Process

Following inhalation anesthesia with isoflurane (Qingdao Orbiepharm Co., Ltd, Qingdao, Shandong, China) in a sterile surgical room, the dorsal skin of the rabbits was shaved and disinfected with alcohol and iodophor. Using 27 G needles, 3 different samples (PLLA microspheres, PLLA-b-PEG microspheres, and irregular PLLA particles, respectively, mixed with sodium hyaluronate gel as a carrier) were bilaterally injected along the spine. Each injection site received a volume of 0.5 mL, ensuring consistent injections at the same locations on each rabbit's back.

#### Tissue Morphological Observation

Under pentobarbital sodium anesthesia, biopsies of the insert and surrounding subcutaneous tissue were conducted after 4, 26, and 52 weeks of injecting the materials. The collected tissues were fixed in 10% neutral-buffered formalin and embedded in paraffin. A vertical microtomy was performed at a thickness of 4 μm. The samples were stained with hematoxylin and eosin (H&E) and Masson's trichrome for assessment using light microscopy.

## RESULTS

### Structural Identification of Different Microspheres

**Figure 1.**
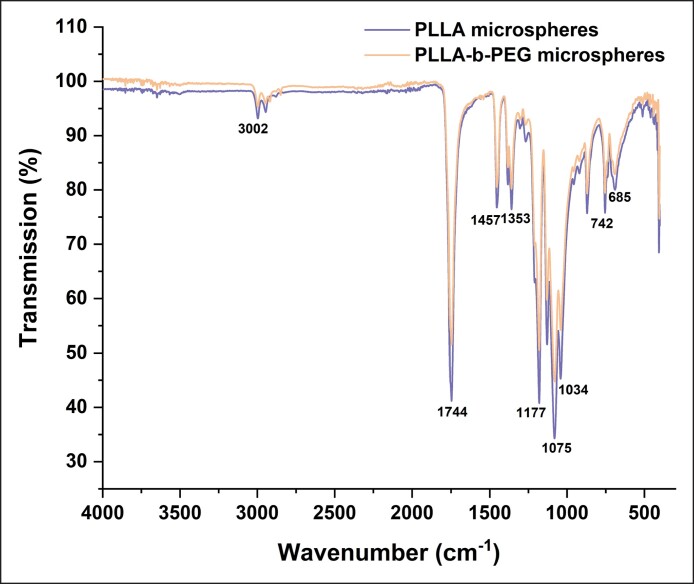
Fourier transform infrared spectroscopy images of PLLA and PLLA-b-PEG microspheres. PEG, polyethylene glycol; PLLA, poly-L-lactic acid.

The chemical structures of PLLA and PLLA-b-PEG microspheres were characterized by FTIR, as shown in Figure 1. The IR spectra of these 2 microspheres exhibit no discernible differences, regardless of the introduction of PEG. The peaks at 1075 and 1177 cm^−1^ represent the C–C and C–O stretching vibrations, and 1744 cm^−1^ refers to the peak of the C=O skeleton stretching vibration.^23^

### Microscopic Morphology Characterization of Different Microspheres

**Figure 2.**
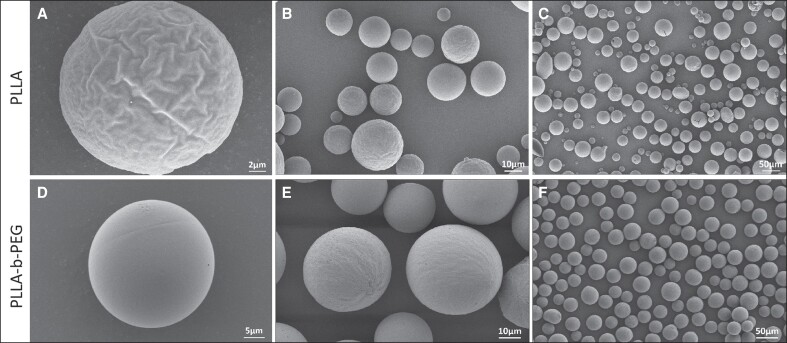
Scanning electron microscopy images of PLLA and PLLA-b-PEG microspheres at different magnifications. (A–C) PLLA microspheres; (D–F) PLLA-b-PEG microspheres. PEG, polyethylene glycol; PLLA, poly-L-lactic acid.

SEM images of the PLLA and PLLA-b-PEG microspheres at different magnifications are shown in Figure 2. All the microspheres were found to be in the range of 20 to 45 μm. It can be seen that there are some differences between these 2 types of microspheres. The morphological features of the microspheres prepared using pure PLLA included an uneven size distribution and a rougher surface with numerous folds, while the surface of PLLA-b-PEG microspheres was smoother without obvious wrinkles.

### In Vitro Degradation Rates of Different Microspheres

**Figure 3.**
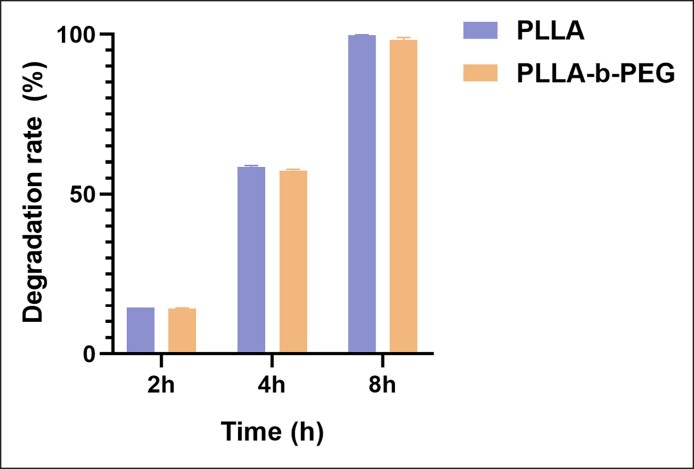
In vitro degradation rates of PLLA and PLLA-b-PEG microspheres at different times. PEG, polyethylene glycol; PLLA, poly-L-lactic acid.

The in vitro degradation rates of the 2 types of microspheres are shown in Figure 3 in the form of a histogram. Both of them had nearly identical degradation rates and were almost completely degraded after 8 h. The in vitro degradation results revealed that the short-chain PEG used in this study did not accelerate the degradation of PLLA.

### Dispersion Uniformity of Microspheres in the Gel

**Figure 4.**
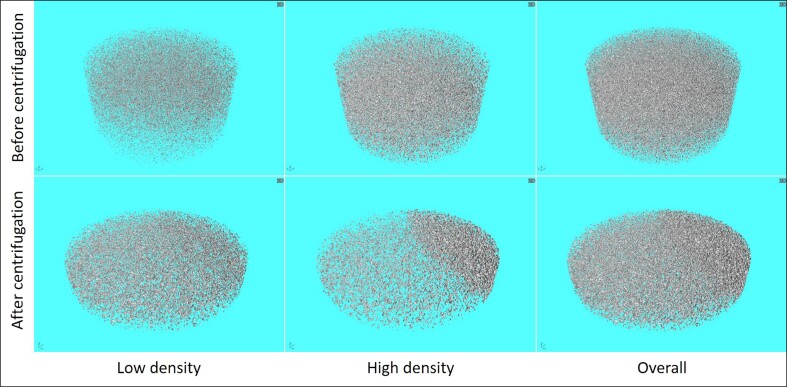
X-ray computed tomography 3-dimensional images of the cross-linked hyaluronic acid gel containing PLLA-b-PEG microspheres before and after centrifugation with different densities. PEG, polyethylene glycol; PLLA, poly-L-lactic acid.

Figure 4 shows the 3-dimensional images of the dispersion of PLLA-b-PEG microspheres in the cross-linked sodium hyaluronate gel, which was determined by X-ray CT. The principle of X-ray CT imaging is based on the different densities between microspheres (high density) and gel (low density). The upper row of this figure illustrates a homogenous dispersion pattern of microspheres within the gel matrix, with the left side representing a low-density region primarily composed of cross-linked sodium hyaluronate gel and the middle column depicting a high-density area mainly consisting of PLLA-b-PEG microspheres. To validate the aforementioned conclusions, we use the gel containing microsphere after centrifugation as a control (bottom row in the figure). The high-density region exhibited a notable concentration of centrifuged microspheres on 1 side of the gel, attributed to the influence of centrifugal force.

### Inflammatory Response and Collagen Growth at the Implant Sites in Rabbit Models

**Figure 5.**
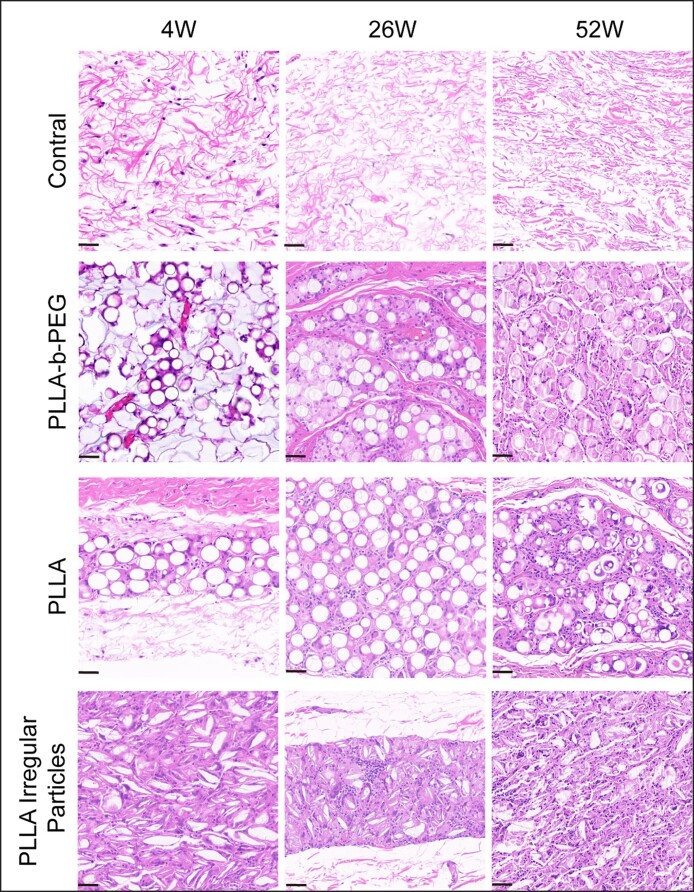
Hematoxylin and eosin staining performed to evaluate the tissue inflammatory response after 4, 26, and 52 weeks of injecting 3 kinds of materials into the dorsal skin of rabbits (×200; scale bar = 50 μm). PEG, polyethylene glycol; PLLA, poly-L-lactic acid.

**Figure 6.**
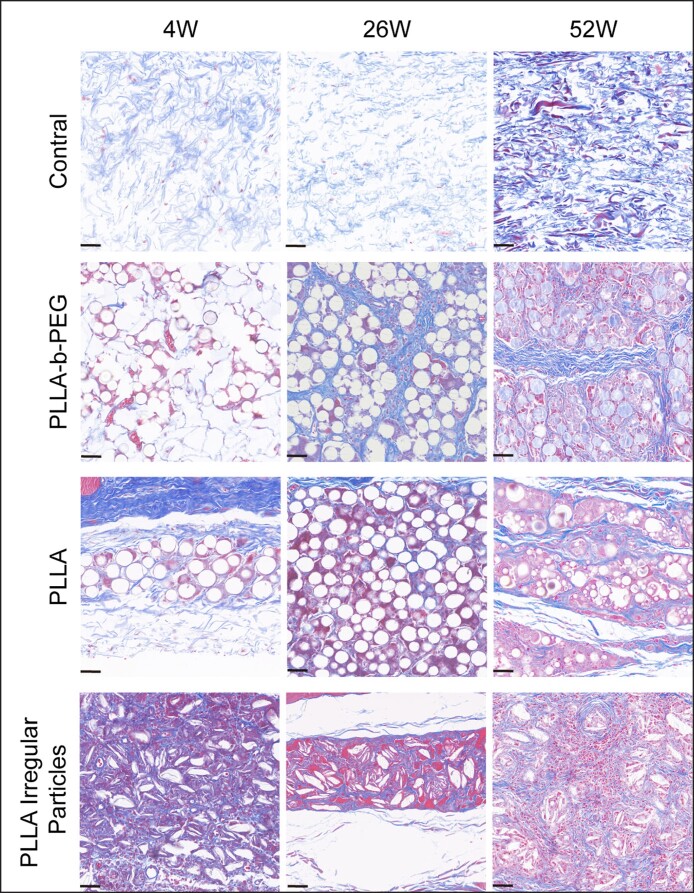
Masson staining performed to evaluate collagen deposition after 4, 26, and 52 weeks of injecting 3 kinds of materials into the dorsal skin of rabbits (×200; scale bar = 50 μm). PEG, polyethylene glycol; PLLA, poly-L-lactic acid.

The histomorphological results of different PLLA with varying microscopic morphology and surface hydrophilicity stained by H&E and Masson are shown in Figures 5 and 6, respectively. In the blank tissue, histological examination revealed a normal skin fiber arrangement with no observable pathological changes in the skin structure. Subsequent implantation in rabbit models demonstrated that PLLA-b-PEG microspheres increased the size of the fibrous envelope and the number of microvessels, fibroblasts, and foreign-body macrophages within the implantation area. These findings suggested the establishment of a well-supported blood supply environment at the implantation site,^24^ providing favorable conditions for collagen production by the clustered fibroblasts. Masson staining further validated these observations, revealing a substantial augmentation in new blue collagen fibers with enhanced collagen organization. Contrarily, PLLA implantation induced a more pronounced early inflammatory response than PLLA-b-PEG, resulting in fewer newly formed collagen fibers and a looser collagen arrangement.

Meanwhile, we also observed the histological behavior of irregular PLLA particles after implantation in vivo. However, the implantation of irregular PLLA particles induced a heightened inflammatory response characterized by prominent macrophages.

## DISCUSSION

PLLA is a biocompatible and biodegradable synthetic implant that acts by stimulating a host response leading to fibroplasia, facilitating gradual volume restoration. It has been utilized in the field of medical aesthetics worldwide for over 2 decades. Numerous studies on PLLA fillers, including the mechanism of action, clinical experiences and consensus, complications and their management, etc, have been extensively discussed in the previous research.^10,17,25-29^

In our previous studies, animal experiments and clinical data demonstrated the efficacy and safety of PLLA-b-PEG microsphere fillers after injection.^30^ However, these studies have some limitations, a lack of comparison of the effect of traditional PLLA and PLLA-b-PEG microspheres, and the research on single-material microsphere fillers is 1 sided. Therefore, on the basis of previous research, this study discussed the differences between PLLA and PLLA-b-PEG microspheres in vitro and in vivo.

Overall, PLLA-b-PEG microspheres exhibited a more gentle stimulation of collagen formation without any significant changes in the physicochemical properties when compared with PLLA microspheres. The IR spectra of the 2 microspheres, which were nearly indistinguishable, indicated their comparable structural characteristics. This similarity could be attributed to the utilization of short chains of PEG block and the lower PEG/PLLA ratio in the block copolymers. The same findings were observed for in vitro degradation rates, with shorter PEG blocks not sufficient to affect the overall polymer degradation curve. Additionally, the presence of short PEG chains ensures that block copolymer microspheres in the body are effectively eliminated through degradation, preventing any excessive residue from accumulating.^31^

However, although the PEG block chain of PLLA-b-PEG was relatively short and not enough to affect the results of IR spectroscopy and in vitro degradation, its introduction improved the morphology of the microspheres and made their surfaces smoother, resulting in a lower inflammatory response and better histocompatibility.^22^ This transformation is closely associated with the hydrophilic nature of PEG, which mitigates the excessive oiliness and viscosity of PLLA while enhancing its hydrophilicity.^32,33^ Uniform cutting in the emulsification stage of the microspheres preparation process also improves the microscopic morphology of the microspheres.^34^ The increased hydrophilicity of the microspheres surface further facilitates homogeneous mixing between the microspheres and the gel, helping the PLLA-b-PEG microspheres to disperse evenly in the hydrogel. This contributes to the uniform distribution of microspheres in vivo, thereby reducing the likelihood of eliciting an inflammatory response and granulomas.^35^

The histopathological analysis also revealed that the collagen formed around PLLA-b-PEG microspheres was denser than PLLA microspheres. PEG modification enhanced the biocompatibility of PLLA materials, and sustained stimulation at a constant intensity yielded healthier collagen with a prolonged residence time.^10,36,37^ This dynamic and orderly collagen deposition can fill facial contour defects and further reduce the occurrence of adverse reactions, such as granuloma formation.^31^ In previous in vivo clinical studies, it has also been reported that granulomas tend to form after PLLA implantation.^38^ However, after the injection of PLLA-b-PEG microspheres, a mild and sustained tissue response was observed without explosive inflammation in the patient's tissue, confirming its safety further. In terms of effectiveness, 90% of patients were considered to be “very much improved” and “much improved” in the follow-up visits after 3 and 12 weeks.^30^

Our bodies can recognize discrepancies in the size and shape of foreign objects, and the presence of small fragmented particles and sharp particle edges elicits a more pronounced inflammatory response,^39^ while smooth spherical particles cause less inflammatory response.^40^ The accumulation of inflammatory cells raises safety concerns because excessive stimulation within a short timeframe induces significant redness and swelling in clinical settings.^13,41^ Although the collagen production of irregular PLLA granules surpassed that of the other 2 implant groups, excessive collagen production was associated with disorganized collagen accumulation, rendering it susceptible to granuloma formation.

In this study, we conducted a comparative analysis of the physicochemical properties and in vivo performance between PLLA and PLLA-b-PEG microspheres to further demonstrate that the collagen-stimulating ability of PLLA remains unaffected and becomes milder after the introduction of PEG hydrophilic fragments. Despite its numerous strengths, this study is not without limitations. First, this study focused on the in vitro properties and animal tissue responses of PLLA and PLLA-b-PEG microspheres, but the clinical data were absent. Local histological studies alone are insufficient to fully observe the comprehensive systemic changes following microspheres injection, and the underlying causes and mechanisms responsible for the differential stimulation of collagen by the 2 types of microspheres remain unexplained from the genomic level or other perspectives. The future studies should consider incorporating quantitative methods in histology and genomics, along with relevant clinical data, to enhance the analysis of the effects of different materials on tissues.

## CONCLUSIONS

In this study, we demonstrated that the addition of hydrophilic short-chain PEG to hydrophobic PLLA had no significant effect on its molecular weight, main chemical structure, and degradation rate. Comparing the PLLA and PLLA-b-PEG microspheres revealed that the presence of PEG afforded microspheres with smoother surfaces. This phenomenon can be attributed to the amphiphilicity of the PLLA-b-PEG, leading to preferential accumulation of the PEG on the surface of the microspheres during the process of oil-in-water emulsion-solvent evaporation. Furthermore, in vivo animal implantation experiments confirmed that PLLA-b-PEG microspheres have a lower inflammatory response and produce firmer and more orderly collagen deposits than pure PLLA microspheres. The lower inflammatory response may be attributed to the more regular and hydrophilic surfaces of PLLA-b-PEG microspheres. However, the impact of PEG on collagen fiber deposition requires further investigation. Future research must be aimed at exploring the impact of PEG on PLLA-b-PEG microspheres, focusing on fabrication optimization, understanding degradation behavior and cellular responses, and assessing the potential for clinical applications.
